# Entanglement and Non-Locality in Quantum Protocols with Identical Particles

**DOI:** 10.3390/e23040479

**Published:** 2021-04-18

**Authors:** Fabio Benatti, Roberto Floreanini, Ugo Marzolino

**Affiliations:** 1 Department of Physics, University of Trieste, 34151 Trieste, Italy; 2 Istituto Nazionale di Fisica Nucleare (INFN), Sezione di Trieste, 34151 Trieste, Italy; Roberto.Floreanini@ts.infn.it (R.F.); Ugo.Marzolino@ts.infn.it (U.M.)

**Keywords:** entanglement theory, identical particles, quantum metrology, interferometry, quantum teleportation

## Abstract

We study the role of entanglement and non-locality in quantum protocols that make use of systems of identical particles. Unlike in the case of distinguishable particles, the notions of entanglement and non-locality for systems whose constituents cannot be distinguished and singly addressed are still debated. We clarify why the only approach that avoids incongruities and paradoxes is the one based on the second quantization formalism, whereby it is the entanglement of the modes that can be populated by the particles that really matters and not the particles themselves. Indeed, by means of a metrological and of a teleportation protocol, we show that inconsistencies arise in formulations that force entanglement and non-locality to be properties of the identical particles rather than of the modes they can occupy. The reason resides in the fact that orthogonal modes can always be addressed while identical particles cannot.

## 1. Introduction

Entanglement is the strongest among quantum correlations, rooted in the structurally non-local behavior of quantum mechanics. It is a fundamental resource in most quantum information protocols and processes, as shown in actual applications using different physical settings, e.g., in quantum optics and atomic and molecular physics [1,2,3,4,5,6].

Although well established for systems of distinguishable particles [7,8], the notions of non-locality and entanglement are challenging in systems of indistinguishable particles, as in this case, particles cannot be individually addressed and measured. On the other hand, many-body systems, made of identical constituents, are central to condensed matter physics and therefore of utmost importance in actual applications. Our aim is to provide instances in which the implementation of quantum protocols with systems of identical particles results in being particularly effective, possibly stimulating a discussion on their actual experimental realization.

Many-body systems are more easily treated by adopting the so-called “second-quantization” approach; within this framework, the notions of non-locality and entanglement valid for distinguishable particles can be easily generalized to hold in systems made of identical constituents, in a way applicable to all physical situations. Although the quantum-enhanced performances of technological tasks can be achieved by exploiting other quantum resources than solely entanglement [9,10], in the following, we focus on quantum protocols that require entanglement and are implemented by means of systems consisting of many identical particles. Specifically, we shall analyze two quantum protocols, one in quantum metrology and the other one in teleportation, and describe in detail how for identical particle systems, non-locality and entanglement can be used to reach accuracies beyond those obtainable with classical methods.

Concerning the first protocol, a paradigmatic quantum metrological task is the estimation of interferometric phases [9,11,12,13,14], which has applications for the detection of gravitational waves [15], angular rotation [16,17,18,19,20], and temperature [21]. As we shall see, the difference between distinguishable and indistinguishable particles is that the former require initial state entanglement to beat classical performances, while the latter achieve quantum enhancements with initial separable states as the same interferometers themselves can generate the necessary indistinguishable particle entanglement [22,23]. Thus, particle indistinguishability mitigates the needed efforts in actual experimental implementations.

On the other hand, the second protocol, quantum teleportation, is known to play a prominent role in quantum computation [24,25,26,27,28,29] and quantum communication [30,31,32,33]. In this respect, a particular application of teleportation is the so-called entanglement swapping, which is the generation of quantum correlations between distant subsystems using their entanglement with an auxiliary system; as discussed below, it can be effectively implemented using identical particle systems.

Let us point out that recent technological advances in quantum optics [3,4,5,6,34,35,36], as well as in ultracold atom physics [37,38,39,40] make these protocols likely in the reach of present experimental abilities, thus paving the way to the adoption of many-body systems, made of indistinguishable constituents, as the standard framework for the actual realization of quantum information tasks. Indeed, identical particle systems offer clear advantages in implementing quantum informational protocols with respect to the more standard realizations using distinguishable qubits. First of all, using systems of singly addressable constituents, one needs to keep them always well localized in order to preserve their distinguishability, and this requires more resources. Moreover, as already mentioned before, in order to get accuracies beyond the classical limit with systems of identical particles, there is no need to initially prepare them in an entangled state before feeding them to an apparatus implementing a specific quantum task.

Although we focus here on the notion of entanglement for indistinguishable particle systems in the second quantized framework (the so-called mode entanglement) [41,42,43,44,45,46,47], different approaches to identical particle entanglement have been proposed in the literature (see [48] for a complete review). They have been introduced having in mind specific physical models and applications to limited quantum tasks; in one way or the other, they fail to provide a comprehensive and at the same time fully consistent theory of identical particle entanglement valid in all situations. Indeed, the different definitions of entanglement, alternative to mode entanglement, may produce contradictory results, e.g., in metrological applications, where beating the classical limit to the reachable accuracy is apparently possible without any entanglement ether in the initial state or generated during the protocol.

On the other hand, mode entanglement [48,49,50], the approach adopted in the present investigation, has been proven to be free of such inconsistencies. As discussed in detail below, in this approach, the subsystems to be entangled are identified with modes in second quantization rather than (unaddressable) particles, a natural approach in quantum optics [3,4], quantum field theory [51,52,53,54,55,56], and quantum statistical mechanics [51].

In the second part of the work, we examine different approaches to indistinguishable particle entanglement alternative to mode entanglement; using established results provided by the literature, we discuss in detail, through explicit examples, how these approaches fail to properly identify quantum resources as a consequence of their inability to consistently cope with the locality issue.

The structure of the paper is as follows. In Section 2, we introduce the theory of mode entanglement, its motivations, techniques, and applications. In Section 3, the two benchmark protocols, i.e., interferometric phase estimation and teleportation, are discussed within the framework of mode entanglement. Then, in Section 4, we compare the predictions relative to the two protocols provided by the mode entanglement point of view with those of some alternative approaches. Conclusions are finally drawn in Section 5, while more technical details are provided in Appendix A and Appendix B.

We do hope that the present investigation will reinforce the attention on the advantages of using many-body systems for performing quantum informational tasks, especially in view of possible experimental realizations.

## 2. Mode Entanglement

The main reason behind the existence of different notions of identical particle entanglement and their inconsistencies is the attempt of most of them to hinge on the particle picture in a context where particles cannot be individually addressed and measured. Clearly, the focus on the particle aspect is prompted by entanglement theory as it has been developed for systems made of distinguishable particles. In order to appreciate the kind of difficulties that arise by pursuing too close an approach to the standard one, let us consider two bosons prepared in two orthogonal states |χ〉 and |ϕ〉. Because of their indistinguishability, we can only say that one boson is in the state vector |χ〉 and the other one in the state vector |ϕ〉 orthogonal to |χ〉, but we cannot attribute to any of them a specific particle label. The corresponding normalized state must indeed be symmetric,

(1) $$|\Psi \rangle =\frac{|\chi \rangle ⊗|\phi \rangle \;+\;|\phi \rangle ⊗|\chi \rangle }{\sqrt{2}}\;,$$

so that neither |χ〉 nor |ϕ〉 can be specifically attributed as a state to a specific particle. In the case of two distinguishable particles, |Ψ〉 would be an entangled state. Is this so also for two bosons?

This question has a counterpart that involves operators. Indeed, bipartite entanglement is strictly related to the notion of the locality of observables. In practice, given two distinguishable particles, labeled by 1 and 2, one deals with observables of Particle 1 of the form X⊗𝟙, belonging to the set $O_{1}$, respectively of Particle 2 of the form 𝟙⊗Y, in the corresponding set $O_{2}$. These two sets of operators are subalgebras of the algebra $O=O_{1}\cup O_{2}$ containing the observables of the two-particle system. In particular, two-particle observables of the following form, i.e., the product of two single-particle operators,

(2) $$\left(X⊗𝟙\right)\left(𝟙⊗Y\right)=X⊗Y\;,$$

is called local; indeed all elements of $O_{1}$ commute with all elements of $O_{2}$, whence local measurements of operators from $O_{1}$ and $O_{2}$ do not influence each other. It turns out that entangled state vectors of the two particles are those |Ψ〉 that violate the factorization of correlation functions of local observables:

(3) 〈Ψ|X⊗Y|Ψ〉≠〈Ψ|X⊗𝟙|Ψ〉〈Ψ|𝟙⊗Y|Ψ〉,

for at least one commuting pair $X\in O_{1}$ and $Y\in O_{2}$. Notice that the equality in the previous expression would witness the statistical independence of the two observables with respect to the given bipartite state vector |Ψ〉. One easily checks that the entanglement of a state as |Ψ〉 in (1) is then witnessed by the local observable $P_{\chi }⊗P_{\phi }$, where $P_{\chi }$, respectively $P_{\phi }$, projects onto |χ〉, respectively |ϕ〉,

$$\langle \Psi |P_{\chi }⊗P_{\phi }|\Psi \rangle =\frac{1}{2}\neq \langle \Psi |P_{\chi }⊗𝟙|\Psi \rangle \;\langle \Psi |𝟙⊗P_{\phi }|\Psi \rangle =\frac{1}{4}\;.$$

In a nutshell, distinguishable particle pure state entanglement can be understood as the ability of certain state vectors to make statistically dependent certain particle observables that are algebraically independent, namely commuting.

Unfortunately, tensor products as X⊗𝟙, 𝟙⊗Y, and X⊗Y cannot be sensible operators for identical particles. Indeed, they attach an operator to a specific particle, *X* to Particle 1 and *Y* to Particle 2, making them distinguishable; in order to make those tensor products meaningful observables for indistinguishable particles, one needs to symmetrize them, yielding:

(4) $$X_{\mathrm{sym}}\equiv X⊗𝟙\;+\;𝟙⊗X\;,\;Y_{\mathrm{sym}}\equiv Y⊗𝟙\;+\;𝟙⊗Y\;,$$

for the single-particle operators and:

(5) C=X⊗Y+Y⊗X,

for the two-particle ones. Only in this way does one avoid the association of a specific particle label either to *X* or *Y*. However, unlike X⊗Y, *C* is non-local from the distinguishable particle point of view. Does the same conclusion hold true for identical particles?

In other words, the question is whether symmetrization, or anti-symmetrization for fermions, automatically generates entanglement and non-locality.

**Remark** **1.** *There is however a further and deeper issue connected with the notion of locality for identical particles when one tries to formulate it in terms of tensor products of single-particle commuting operators. Although the single-particle operators $X_{\mathrm{sym}}$ and $Y_{\mathrm{sym}}$ commute if and only if [X,Y]=0, their products yield operators of the form C in *(5)* if and only if XY=0. There are thus plenty of two-particle observables that one would deem local, but cannot be expressed as products of single-particle observables. Such an observation is at the root of the fact that identical particle locality cannot be consistently formulated hinging upon the notion of particles and upon the ensuing description in terms of tensor products either of states or of operators [48]. A satisfactory way out of this puzzle, as well as the answers to the two questions raised above come as follows.*

Though identical particles can be addressed within the particle-based formalism of first quantization, which labels particle degrees of freedom as exemplified in (1), the most natural tools for describing the physics of many-body systems are those of second quantization. In second quantization, one focuses on modes, namely single-particle state vectors ψ, and on their creation $a_{\psi }^{†}$ and annihilation operators $a_{\psi }$, instead of on the particles themselves. Acting on the vacuum |vac〉, such that $a_{\psi }|\mathrm{vac}\rangle =0$, $a_{\psi }^{†}$ creates a particle in the state |ψ〉, while its adjoint $a_{\psi }$ destroys a particle in the state ψ:

(6) $$a_{\psi }^{†}|\mathrm{vac}\rangle =|\psi \rangle \;,\;a_{\psi }|\psi \rangle =|\mathrm{vac}\rangle \;.$$

These operators satisfy the canonical commutation relations for bosons:

(7) $$\left[a_{\psi },a_{\varphi }^{†}\right]\equiv a_{\psi }\;a_{\varphi }^{†}\;-\;a_{\varphi }^{†}\;a_{\psi }=\langle \psi |\varphi \rangle \;,$$

and anti-commutation relations for fermions,

(8) $$\left\{a_{\psi },a_{\varphi }^{†}\right\}\equiv a_{\psi }\;a_{\varphi }^{†}\;+\;a_{\varphi }^{†}\;a_{\psi }=\langle \psi |\varphi \rangle \;.$$

Creation and annihilation operators are the building blocks for constructing firstly polynomials, involving products of powers of them, and eventually generic functions of them forming suitable algebras of operators.

Given two orthogonal subsets $H_{1,2}$ of orthonormal modes $\{|\psi_{j}\rangle {\}}_{j=1}^{n_{1}}$ and $\{|\varphi_{k}\rangle {\}}_{k=1}^{n_{2}}$, we shall denote by $A_{1,2}$ the algebras generated by the creation and annihilation operators ${\left\{a_{\psi_{j}},a_{\psi_{j}}^{†}\right\}}_{j=1}^{n_{1}}$, respectively ${\left\{a_{\varphi_{k}},a_{\varphi_{k}}^{†}\right\}}_{k=1}^{n_{2}}$. From the commutation relations, it follows that any pair of elements $A_{1}\in A_{1}$, $A_{2}\in A_{2}$ commutes: $[A_{1},A_{2}]=0$. Having abandoned the use of the tensor product structure inherent in the particle approach, one can now consistently extend to systems of identical particles the characterization of entanglement through the lack of factorization of two point correlation functions for local operators as in (5). In this way, focusing on operator commutativity, one first generalizes:

1. the notion of locality for distinguishable particles to mode-locality for identical particle systems by declaring local any product:
  (9) $$A_{12}\;=\;A_{1}A_{2}\;,\;A_{1}\in A_{1}\;,\;A_{2}\in A_{2}\;;$$
  and then:
2. the notion of bipartite entanglement for pure states, by declaring entangled those state vectors |Ψ〉 for which:
  (10) $$\langle \Psi |A_{1}A_{2}|\Psi \rangle \;\neq \;\langle \Psi |A_{1}\left|\Psi \rangle \;\langle \Psi \right|A_{2}|\Psi \rangle \;,$$
  for at least one couple $A_{1}$, $A_{2}$ of local operators.

We shall refer to the pair of commuting algebras $\left(A_{1},A_{2}\right)$ as an algebraic bipartition; certainly, both mode-locality and mode entanglement depend on the choice of bipartition, and the possible bipartitions are uncountably many, unlike in the standard distinguishable particle setting where $A_{1}$ and $A_{2}$ are essentially fixed to be the algebras $O_{1}$ and $O_{2}$ of the observables of Particle 1 and of Particle 2. Furthermore, it turns out (see [48] and the references therein) that a state vector is separable, that is non-entangled, with respect to a chosen algebraic bipartition $\left(A_{1},A_{2}\right)$, if and only if:

(11) $$|\Psi_{\mathrm{sep}}\rangle =f_{1}^{†}f_{2}^{†}\;|\mathrm{vac}\rangle \;,$$

where $f_{1,2}^{†}$ are functions of the creation operators ${\left\{a_{\psi_{j}}^{†}\right\}}_{j=1}^{n_{1}}$ in $A_{1}$, respectively ${\left\{a_{\varphi_{k}}^{†}\right\}}_{k=1}^{n_{2}}$ in $A_{2}$. The operators $f_{1,2}$ are chosen such that $\langle \mathrm{vac}|f_{1}\;f_{1}^{†}|\mathrm{vac}\rangle =\langle \mathrm{vac}|\;f_{2}\;f_{2}^{†}|\mathrm{vac}\rangle =1$, whence:

(12) $$\begin{array}{ccc} \langle \Psi_{\mathrm{sep}}|\Psi_{\mathrm{sep}}\rangle & = & \langle \mathrm{vac}|f_{2}\;f_{1}\;f_{1}^{†}\;f_{2}^{†}|\mathrm{vac}\rangle =\langle \mathrm{vac}|\;f_{1}\;f_{1}^{†}\;f_{2}\;f_{2}^{†}|\mathrm{vac}\rangle \\ & = & \langle \mathrm{vac}|f_{1}\;f_{1}^{†}|\mathrm{vac}\rangle \;\langle \mathrm{vac}|\;f_{2}\;f_{2}^{†}|\mathrm{vac}\rangle =1\;. \end{array}$$

**Remark** **2.** *Notice that, when the total number of bosons is fixed, the number of particles in $f_{1,2}^{†}|\mathrm{vac}\rangle$ is also fixed. Hence, the state vectors that are eigenstates of the total boson number and are separable with respect to the bipartition $\left(A_{1},A_{2}\right)$ must also be eigenstates of the number operators:*

(13) N1=∑j=1n1aψj†aψj†,N2=∑k=1n2aφk†aφk†

*counting the bosons in the modes that identify the commuting algebras $A_{1,2}$.*

In the context of mode entanglement, the two questions raised above have a definite answer; indeed, in the second quantization formalism, the state in (1) reads:

(14) $$|\Psi \rangle =a_{\chi }^{†}\;a_{\phi }^{†}\;|\mathrm{vac}\rangle \;,$$

and, according to (11), is thus separable with respect to an algebraic bipartition $\left(A_{1},A_{2}\right)$ with $f_{1}^{†}=a_{\chi }^{†}$ and $f_{2}^{†}=a_{\phi }^{†}$ where $a_{\chi },a_{\chi }^{†}\in A_{1}$ and $a_{\phi },a_{\phi }^{†}\in A_{2}$.

Furthermore, once an orthonormal set $\{|\chi_{j}\rangle {\}}_{j}$ of modes in the single-particle Hilbert space is given, the single-particle observables:

$$X=\sum_{i,j}X_{ij}\;|\chi_{i}\rangle \langle \chi_{j}|\;,\;Y=\sum_{k,\ell }Y_{k\ell }\;|\chi_{k}\rangle \langle \chi_{\ell }|\;,$$

in (5) have the following second quantized expressions (see (4)):

$$X_{\mathrm{sym}}=\sum_{i,j}X_{ij}\;a_{\chi_{i}}^{†}a_{\chi_{j}}\;,\;Y_{\mathrm{sym}}=\sum_{k,\ell }Y_{k\ell }\;a_{\chi_{k}}^{†}a_{\chi_{\ell }}\;.$$

Then, if $X_{\mathrm{sym}}$ belongs to the algebra $A_{1}$ corresponding to the set of modes $\{|\psi_{j}\rangle {\}}_{j=1}^{n_{1}}$ and $Y_{\mathrm{sym}}$ to the algebra $A_{2}$ relative to the set of modes $\{|\varphi_{k}\rangle {\}}_{k=1}^{n_{2}}$, namely,

(15) $$X_{\mathrm{sym}}=\sum_{i,j=1}^{n_{1}}X_{ij}\;a_{\psi_{i}}^{†}a_{\psi_{j}}\;,\;X_{\mathrm{sym}}=\sum_{k,\ell =1}^{n_{2}}Y_{k\ell }\;a_{\varphi_{k}}^{†}a_{\varphi_{\ell }}\;,$$

the symmetrized observable *C* in (5) is local with respect to $\left(A_{1},A_{2}\right)$; indeed, from the commutativity of $A_{1}$ and $A_{2}$, it follows that:

(16) $$\begin{array}{cc} C & =\sum_{i,j=1}^{n_{1}}\sum_{k,\ell =1}^{n_{2}}\;{\left(X⊗Y\right)}_{ik,j\ell }\;a_{\psi_{i}}^{†}\;a_{\varphi_{k}}^{†}\;a_{\varphi_{\ell }}\;a_{\psi_{j}}\\ \; & =\left(\sum_{i,j=1}^{n_{1}}X_{ij}\;a_{\psi_{i}}^{†}\;a_{\psi_{j}}\right)\;\left(\sum_{k,\ell =1}^{n_{2}}Y_{k\ell }\;a_{\varphi_{k}}^{†}\;a_{\varphi_{\ell }}\right)\;. \end{array}$$

It is also worth noting that, according to (9) and (10), mode-separable state vectors $|\Psi_{\mathrm{sep}}\rangle$ with respect to an algebraic bipartition $\left(A_{1},A_{2}\right)$ as in (11) remain separable under the action of mode-local operations transforming $|\Psi_{\mathrm{sep}}\rangle$ into:

(17) $$|\tilde{\Psi }\rangle :=O_{1}O_{2}|\Psi_{\mathrm{sep}}\rangle \;,\;O_{1,2}\in A_{1,2}\;,$$

where $|\Psi_{\mathrm{sep}}\rangle$ is normalized as in (12), while $O_{1}$ and $O_{2}$ are suitably rescaled to satisfy $\langle \Psi_{\mathrm{sep}}|O_{1}^{†}O_{1}|\Psi_{\mathrm{sep}}\rangle =\langle \Psi_{\mathrm{sep}}|O_{2}^{†}O_{2}|\Psi_{\mathrm{sep}}\rangle =1$. Indeed, the form (11) of separable states is maintained by the action of $O_{1}O_{2}$; moreover, for arbitrary local observables $A_{1}A_{2}$, with $A_{1,2}\in A_{1,2}$, setting ${\tilde{A}}_{1,2}=O_{1,2}^{†}A_{1,2}O_{1,2}$, from the fact that $O_{1}^{†}O_{2}^{†}A_{1}A_{2}O_{2}O_{1}=O_{1}^{†}A_{1}O_{1}\;O_{2}^{†}A_{2}O_{2}$, using separability and the normalization conditions, one derives:

(18) $$\begin{array}{cc} \; & \langle \tilde{\Psi }|A_{1}A_{2}|\tilde{\Psi }\rangle =\langle \Psi_{\mathrm{sep}}|{\tilde{A}}_{1}{\tilde{A}}_{2}|\Psi_{\mathrm{sep}}\rangle =\langle \Psi_{\mathrm{sep}}|{\tilde{A}}_{1}|\Psi_{\mathrm{sep}}\rangle \;\langle \Psi_{\mathrm{sep}}|{\tilde{A}}_{2}|\Psi_{\mathrm{sep}}\rangle \\ \; & =\langle \Psi_{\mathrm{sep}}|{\tilde{A}}_{1}|\Psi_{\mathrm{sep}}\rangle \;\langle \Psi_{\mathrm{sep}}|O_{2}^{†}O_{2}|\Psi_{\mathrm{sep}}\rangle \;\langle \Psi_{\mathrm{sep}}|{\tilde{A}}_{2}|\Psi_{\mathrm{sep}}\rangle \;\langle \Psi_{\mathrm{sep}}|O_{1}^{†}O_{1}|\Psi_{\mathrm{sep}}\rangle \\ \; & =\langle \Psi_{\mathrm{sep}}|{\tilde{A}}_{1}\;O_{2}^{†}O_{2}|\Psi_{\mathrm{sep}}\rangle \;\langle \Psi_{\mathrm{sep}}|{\tilde{A}}_{2}\;O_{1}^{†}O_{1}|\Psi_{\mathrm{sep}}\rangle =\langle \tilde{\Psi }|A_{1}|\tilde{\Psi }\rangle \;\langle \tilde{\Psi }|A_{2}|\tilde{\Psi }\rangle \;. \end{array}$$

**Remark** **3.** *The formulation of mode entanglement in Fermionic systems and the role of canonical anti-commutation relations were discussed in [18,57,58,59,60]. In this case, the parity ${\left(-1\right)}^{N}$, where N is the total number of fermions, is a conserved quantity, and so, observable subalgebras can be decomposed into an even and an odd part, which project N onto the subspace with even and odd eigenvalues, respectively. The odd part of the subalgebras, $A_{1}$ and $A_{2}$, must anti-commute with each other, while the even part of each subalgebra must commute with both the even and the odd part of the other subalgebra. The definitions of mode-local operators *(9)* and of mode-entangled pure states *(10)*, as well as their general expression *(11)* remain unchanged: of course, Fermionic systems are constrained to have at most one particle per mode.*

*Furthermore, mode entanglement can be extended to mixed states [48], thereby generalizing Werner’s reformulation of entanglement for distinguishable particles [7], i.e., Equation *(3)*, where, as already stressed, algebras are made of single-particle operators. Mode entanglement recovers Werner’s definition when modes are occupied by at most one particle. Notice that other existing definitions of indistinguishable particle entanglement are not compatible with Werner’s formulation [48,50]. Indeed, for any choice of subalgebras, $A_{1}$ and $A_{2}$, there are operators $A_{j}\in A_{j}$, j=1,2, and pure states termed separable within these approaches that do exhibit correlations of the type embodied by Inequality *(10)*.*

### Theoretical Setting

In the following, we consider a simplified, yet rather illustrative setting consisting of bosons with spatial S=L,R and internal, σ=↑,↓, degrees of freedom corresponding to the orthonormal basis:

(19) |L,↑〉,|L,↓〉,|R,↑〉,|R,↓〉,

in the single-particle Hilbert space. One can then consider the corresponding mode creation and annihilation operators $a_{S,\sigma }$, $a_{S,\sigma }^{†}$ and proceed to bipartite the whole Bose algebra constructed upon such mode operators into commuting algebras made of functions of the left or of the right mode operators respectively, denoted, in a compact notation, by:

(20) $$A_{L}={\left\{a_{L,\sigma },a_{L,\sigma }^{†}\right\}}_{\sigma =↑,↓}\;,\;A_{R}={\left\{a_{R,\sigma },a_{R,\sigma }^{†}\right\}}_{\sigma =↑,↓}\;.$$

Products $A_{L}A_{R}$ of observables $A_{L}\in A_{L}$ and $A_{R}\in A_{R}$ such as, for instance, the boson number operators,

(21) $$N_{L}=\sum_{\sigma =↑,↓}a_{L,\sigma }^{†}a_{L,\sigma }\;,\;N_{R}=\sum_{\sigma =↑,↓}a_{R,\sigma }^{†}a_{R,\sigma }\;,$$

are mode-local with respect to the algebraic bipartition $\left(A_{L},A_{R}\right)$.

Other choices of commuting algebras, e.g., $A_{↑}={\left\{a_{S,↑},a_{S,↑}^{†}\right\}}_{S=L,R}$ and $A_{↓}={\left\{a_{S,↓},a_{S,↓}^{†}\right\}}_{S=L,R}$, give rise to different kinds of mode entanglement. In all cases, unlike particles that cannot in general be individually addressed, modes described by commuting algebras can.

Though reduced to a few degrees of freedom, the above setting allows one to discuss the usefulness for the quantum metrology and teleportation of *N* bosons occupying the states |L,↑〉 and |R,↓〉. These *N*-boson states belong to a subspace spanned by state vectors that, in first quantization, read:

(22) $$S\;\left({|L,↑\rangle }^{⊗\ell }⊗{|R,↓\rangle }^{⊗(N-\ell )}\right),$$

where S is the symmetrization projector and, in second quantization, become Fock number states:

(23) $$|\Psi_{\mathrm{Fock}}\rangle =\frac{{\left(a_{L,↑}^{†}\right)}^{\ell }}{\sqrt{\ell !}}\;\frac{{\left(a_{R,↓}^{†}\right)}^{N-\ell }}{\sqrt{(N-\ell )!}}\;|\mathrm{vac}\rangle ,$$

satisfying (see (21)):

(24) $$N_{L}|\Psi_{\mathrm{Fock}}\rangle =\ell \;|\Psi_{\mathrm{Fock}}\rangle \;,\;N_{R}\;|\Psi_{\mathrm{Fock}}\rangle =\left(N-\ell \right)\;|\Psi_{\mathrm{Fock}}\rangle \;.$$

A generic normalized state vector spanned by the above Fock states reads:

(25) $$|\Psi \rangle =\sum_{\ell =0}^{N}\alpha_{\ell }\;\frac{{\left(a_{L,↑}^{†}\right)}^{\ell }}{\sqrt{\ell !}}\;\frac{{\left(a_{R,↓}^{†}\right)}^{N-\ell }}{\sqrt{(N-\ell )!}}\;|\mathrm{vac}\rangle \;,\;\alpha_{\ell }\in C\;,\;\sum_{\ell =0}^{N}{\left|\alpha_{\ell }\right|}^{2}=1\;.$$

Particular instances of these states can be manipulated and used as resources for quantum protocols; indeed, we propose to focus on the following states as they exemplify several resource states used in quantum information tasks:

1. a superposition of two Fock number states,
  (26) $$\begin{array}{ccc} |\Psi_{\ell_{1},\ell_{2}}\rangle & = & \sqrt{\xi }\;\frac{{\left(a_{L,↑}^{†}\right)}^{\ell_{1}}}{\sqrt{\ell_{1}!}}\;\frac{{\left(a_{R,↓}^{†}\right)}^{N-\ell_{1}}}{\sqrt{(N-\ell_{1})!}}\;|\mathrm{vac}\rangle \\ \; & + & e^{i\varphi }\sqrt{1-\xi }\;\frac{{\left(a_{L,↑}^{†}\right)}^{\ell_{2}}}{\sqrt{\ell_{2}!}}\;\frac{{\left(a_{R,↓}^{†}\right)}^{N-\ell_{2}}}{\sqrt{(N-\ell_{2})!}}\;|\mathrm{vac}\rangle \;, \end{array}$$
  with 0≤ξ≤1, that reduces to the so-called NOONstate when $\ell_{1}=N-\ell_{2}=0$ and ξ=1/2;
2. a linear superposition of Fock states with uniform weights $\frac{1}{N+1}$ and arbitrary phases,
  (27) $$|\Psi_{\mathrm{unif}}\rangle =\sum_{\ell =0}^{N}\frac{e^{i\varphi_{\ell }}}{\sqrt{N+1}}\;\frac{{\left(a_{L,↑}^{†}\right)}^{\ell }}{\sqrt{\ell !}}\;\frac{{\left(a_{R,↓}^{†}\right)}^{N-\ell }}{\sqrt{(N-\ell )!}}\;|\mathrm{vac}\rangle \;;$$
3. a coherent state of the SU(2) group generated by the two modes (L,↑) and (R,↓),
  (28) $$|\Psi_{\mathrm{coh}}\rangle =\frac{1}{\sqrt{N!}}{\left(\sqrt{\xi }\;a_{L,↑}^{†}+e^{i\varphi }\sqrt{1-\xi }\;a_{R,↓}^{†}\right)}^{N}|\mathrm{vac}\rangle \;,\;0\leq \xi \leq 1\;.$$

With the exception of the Fock number state $|\Psi_{\mathrm{Fock}}\rangle$, which, according to (11), is mode separable with respect to the bipartitions $\left(A_{L},A_{R}\right)$ and $\left(A_{↑},A_{↓}\right)$, all other states are mode entangled with respect to them. Moreover, the state $|\Psi_{\mathrm{unif}}\rangle$ is maximally entangled as quantified by several entanglement measures, like entanglement entropy and entanglement negativity [61,62].

## 3. Two Quantum Protocols

In the following, we study the resourcefulness of the three states introduced above, together with the Fock ones, with respect to two quantum tasks implementable in an identical particle context; the first task is a metrological one and the second a mode-teleportation one. For both tasks, we study how mode entanglement provides a quantum advantage in the first case and enables the implementation of the second one.

### 3.1. Identical Particle Metrology

As a first protocol, let us consider the estimation of the phase-shift θ implemented by a Mach–Zehnder interferometer acting on an *N*-boson state |Ψ〉 as in (25) by shifting the relative phase of the modes |L,↑〉 and |R,↓〉. The action of such an interferometer is described by the unitary operator:

(29) $$U_{\theta }=e^{i\theta \;N_{LR}}\;,\;N_{LR}:=N_{L,↑}-N_{R,↓}\;,$$

where $N_{L,↑}=a_{L,↑}^{†}a_{L,↑}$ and $N_{R,↓}=a_{R,↓}^{†}a_{R,↓}$ count the number of bosons in the modes |L,↑〉 and |R,↓〉.

In quantum metrology, the quantum Cramér–Rao inequality lower bounds the best accuracy $\delta^{2}\theta$ reachable in the determination of θ by the inverse of the quantum Fisher information $F_{|\Psi \rangle }\left(N_{LR}\right)$ relative to the input state |Ψ〉 and the generator $N_{LR}$ of the transformation [11,12,13,14]. The input state being pure, the quantum Fisher information reduces to (four times) the variance $\Delta_{|\Psi \rangle }N_{LR}$ of the generator with respect to the state |Ψ〉. Then:

(30) $$\delta^{2}\theta ⩾\frac{1}{F_{|\Psi \rangle }\left(N_{LR}\right)}=\frac{1}{4\;\Delta_{|\Psi \rangle }N_{LR}}\;,\;\Delta_{|\Psi \rangle }N_{LR}=\langle \Psi |N_{LR}^{2}|\Psi \rangle \;-\;\langle \Psi |N_{LR}{|\Psi \rangle }^{2}\;.$$

The best accuracy achievable by classical interferometry is the inverse of the classical Fisher information; it corresponds to the so-called shot-noise, namely to the Fisher information increasing linearly with the number of particles. Whenever the Fisher information exceeds the shot-noise threshold, quantum features, either in the initial state or in the interferometer, need to be at work.

In the case of the metrological task depicted above, the required quantumness is the mode entanglement of the state with respect to the bipartition $\left(A_{L},A_{R}\right)$. In fact, according to (9), the unitary operator $U_{\theta }$ in (29) is mode-local with respect to the bipartition $\left(A_{L},A_{R}\right)$, for $N_{L,↑}$ and $N_{R,↓}$ commute so that $U_{\theta }$ splits into a product of commuting unitary operators:

(31) $$U_{\theta }=e^{i\theta N_{L,↑}}\;e^{-i\theta N_{R,↓}},\;e^{i\theta N_{L,↑}}\in A_{L}\;,\;e^{-i\theta N_{R,↓}}\in A_{R}\;.$$

It follows that mode entanglement in the initial state is necessary to beat the shot-noise, if the interferometer is mode-local and the particle number is conserved [63,64]. This fact can be best appreciated as follows: if the *N*-boson state |Ψ〉 were mode-separable with respect to $\left(A_{L},A_{R}\right)$, it would be of the form (11) with $f_{1}^{†}=f_{L}^{†}$ a suitable function of $a_{L,↑}^{†}$ and $a_{L,↓}^{†}$ and $f_{2}^{†}=f_{R}^{†}$ a suitable function of $a_{R,↑}^{†}$ and $a_{R,↓}^{†}$. Then, according to Remark 2 and using (12), one computes:

(32) $$\langle \Psi |N_{LR}^{2}|\Psi \rangle =\langle f_{L}|N_{L↑}^{2}|f_{L}\rangle \;+\;\langle f_{R}|N_{R↓}^{2}|f_{R}\rangle \;-\;2\;\langle f_{L}|N_{L↑}|f_{L}\rangle \;\langle f_{R}|N_{R↓}|f_{R}\rangle \;,$$

where $|f_{L,R}\rangle \equiv f_{L,R}^{†}|\mathrm{vac}\rangle$. It thus follows that:

(33) $$F_{|\Psi \rangle }\left(N_{LR}\right)=4\;\Delta_{|\Psi \rangle }N_{LR}=4\;\Delta_{|f_{L}\rangle }N_{L,↑}+4\;\Delta_{|f_{R}\rangle }N_{R,↑}=0\;.$$

Indeed, since the total number of bosons is fixed to be *N*, $|f_{L}\rangle$ and $|f_{R}\rangle$ are as $|\Psi_{\mathrm{Fock}}\rangle$ in (23), hence eigenstates of the number operators $N_{L,↑}$ and $N_{R,↓}$. The vanishing variances are in agreement with the fact that phase-changes and particle number operators are conjugate observables so that perfect knowledge of one of them entails total ignorance about the other one. This same situation occurs with any fixed, finite number of modes, or for distinguishable particles with a fixed and finite single-particle Hilbert space dimension.

For the mode-entangled states in (25), one finds:

(34) $$N_{LR}|\Psi \rangle =\sum_{\ell =0}^{N}\left(2\ell -N\right)\;\alpha_{\ell }\;\frac{{\left(a_{L,↑}^{†}\right)}^{\ell }}{\sqrt{\ell !}}\;\frac{{\left(a_{R,↓}^{†}\right)}^{N-\ell }}{\sqrt{(N-\ell )!}}\;|\mathrm{vac}\rangle \;,$$

whence (34) together with (30) yields:

(35) $$F_{|\Psi \rangle }\left(N_{LR}\right)=4\sum_{\ell =0}^{N}{\left|\alpha_{\ell }\right|}^{2}\;\ell^{2}-4{\left(\sum_{\ell =0}^{N}{\left|\alpha_{\ell }\right|}^{2}\;\ell \right)}^{2}\;.$$

In the case of the specific states in (26)–(28), the Fisher information reads:

(36) $$\begin{array}{cc} F_{|\Psi_{\ell_{1},\ell_{2}}\rangle }\left(N_{LR}\right)= & 4\;\xi \left(1-\xi \right){\left(\ell_{1}-\ell_{2}\right)}^{2}, \end{array}$$

(37) $$\begin{array}{cc} F_{|\Psi_{\mathrm{unif}}\rangle }\left(N_{LR}\right)= & \frac{1}{3}\left(N^{2}+2N\right) \end{array}$$

(38) $$\begin{array}{cc} F_{|\Psi_{\mathrm{coh}}\rangle }\left(N_{LR}\right)= & 4\;\xi (1-\xi )N\;. \end{array}$$

It follows that $F_{|\Psi_{\ell_{1},\ell_{2}}\rangle }\left(N_{LR}\right)$ beats the shot-noise whenever $F_{|\Psi_{\ell_{1},\ell_{2}}\rangle }\left(N_{LR}\right)>N$, e.g., for the NOON state when $\ell_{1}=N-\ell_{2}=0$ and ξ=1/2. In this case, $F_{|\Psi_{0,N}\rangle }\left(N_{LR}\right)=N^{2}$, reaching the maximum value for the quantum Fisher information, the so-called Heisenberg limit. As for the other states, $F_{|\Psi_{\mathrm{unif}}\rangle }\left(N_{LR}\right)$ always beats the shot-noise: indeed, if the phases $\varphi_{\ell }\propto \ell$, $|\Psi_{\mathrm{unif}}\rangle$ is the eigenstate of the phase operator canonically conjugated to $N_{LR}$ [65]. Instead, $F_{|\Psi_{\mathrm{coh}}\rangle }\left(N_{LR}\right)$ never beats the shot-noise because $|\Psi_{\mathrm{coh}}\rangle$ is a coherent state of the SU(2) group whose elements model linear interferometers and reproduce classical performances. In particular, while Equation (33) shows that entanglement is necessary if the particle number is conserved [63,64], Equation (38) implies that entanglement is not in general sufficient to beat the shot-noise with local interferometers. It is indeed well known also for distinguishable particles that not all entangled states are useful for quantum metrological advantages.

Now, let us consider the unitary operator:

(39) $$V_{\theta }:=e^{i\;\theta \;T_{LR}}\;,\;T_{LR}:=a_{L,↑}^{†}a_{R,↓}+a_{R,↓}^{†}a_{L,↑}\;.$$

It describes a transformation that is non-local with respect to the bipartition $\left(A_{L},A_{R}\right)$ and amounts to a phase-change operated through a rotation of the modes (L,↑) and (R,↓), implementable by means of beam splitters. By acting with $V_{\theta }$ on the state $|\Psi_{\mathrm{Fock}}\rangle$ in (23), which we have seen to be mode-separable with respect to the bipartition $\left(A_{L},A_{R}\right)$, one can beat the shot-noise limit when ℓ≠0,N [22,23]. Indeed, one computes:

(40) $$F_{|\Psi_{\mathrm{Fock}}\rangle }\left(T_{LR}\right)=4\;\left(\langle \Psi_{\mathrm{Fock}}|T_{LR}^{2}|\Psi_{\mathrm{Fock}}\rangle -\langle \Psi_{\mathrm{Fock}}|T_{LR}{|\Psi_{\mathrm{Fock}}\rangle }^{2}\right)=N+2\ell \left(N-\ell \right)\;,$$

which improves upon the shot-noise at least by a multiplicative constant for ℓ≠0,N, while reaching the Heisenberg scaling $F_{|\Psi_{\mathrm{Fock}}\rangle }\left(T_{LR}\right)∼N^{2}/2$ if ℓ∼N/2. Instead, when ℓ=0,N, the state $|\Psi_{\mathrm{Fock}}\rangle$ is of the coherent form of $|\Psi_{\mathrm{coh}}\rangle$ in (28) with ξ=0,1, which indeed ensures only classical performances. On the other hand, if ℓ≠0,N, the resource able to provide the entanglement in the output state necessary for quantum-enhanced performances is the non-local action of the interferometer. It is therefore possible to achieve quantum advantages with mode-separable states of *N* particles independently prepared in two non-overlapping localizations and internal states. Namely, there is no need to entangle the input state if the devices operates non-locally on the modes being used. Indeed, the theory of mode entanglement identifies the entangling power of beam splitters as a quantum resource [64,66,67], as also happens with violations of Bell’s inequalities in similar physical situations [68,69].

### 3.2. Mode Teleportation Protocol

As well known, quantum teleportation [70] is a protocol for the transmission of an unknown quantum state to a remote location using an entangled resource state, local operations, and classical communication. In the standard framework of distinguishable particles, states of a specific particle, say particle *A*, are teleported to a distant one, say particle *C*. This latter particle is initially entangled with another particle, say particle *B*, that is close to particle *A*. The sender performs a projective measurement onto Bell states of the particles *A* and *B*; then, according to the obtained result, which is sent to him/her through a classical communication channel, the receiver applies a local operation to particle *C*, whose state finally turns out to coincide with the initial state of particle *A*. This protocol has been generalized to three indistinguishable particles by considering a fully symmetrized or antisymmetrized state [71], whereby it was shown that symmetrization or antisymmetrization cannot consistently generate entanglement useful for implementing teleportation.

In many-body systems, indistinguishable particles are not in general confined to different locations, otherwise they could be distinguished, neither can they be individually addressed, e.g., by ascribing them unambiguous properties. A full generalization of the teleportation protocol in a many-body context consists of teleporting superpositions of the occupation number eigenstates of suitable sets of modes in the Fock space, rather than particle states as done in a first quantization approach [72,73,74,75]. This general protocol recovers the standard teleportation protocols involving distinguishable particles when the local particle occupation numbers of these modes are fixed. Indeed, in this case, addressing modes coincides with addressing particles.

In order to be more specific, consider a state of *M* indistinguishable bosons distributed between the two modes (X,↑) and (Y,↓) with spatial locations *X* and *Y*, according to the state:

(41) $$|\Phi \rangle =\sum_{\ell =0}^{M}c_{\ell }\;\frac{{\left(a_{X,↑}^{†}\right)}^{\ell }}{\sqrt{\ell !}}\;\frac{{\left(a_{Y,↓}^{†}\right)}^{M-\ell }}{\sqrt{(M-\ell )!}}\;|\mathrm{vac}\rangle \;,$$

where the mode (Y,↓) plays the role of particle *A* in the standard setting. The goal of the protocol presented below is to teleport particle states from the mode (Y,↓) to the mode (R,↓), which then corresponds to particle *C* in the standard setting. Such a mode is accessible to *N* bosons described by a resource state |Ψ〉 as in Equation (25) that is thus mode-entangled with respect to the algebraic bipartition $\left(A_{L},A_{R}\right)$. In this scheme, the remaining mode (L,↑) plays thus the role of particle *B* in standard teleportation.

The protocol consists of:

1. a projective measurement onto Bell-like states with fixed particle numbers relative to the modes (Y,↓) and (L,↑) (see Appendix A), assuming that, as particles *A* and *B* in the standard protocol, the location *L* is close enough to the location *Y* to allow for coherent manipulations of states;
2. a local operation on the mode (R,↓) conditioned to the Bell measurement outcome that needs to be communicated between the locations *L* and *R* using classical devices.

The operations involved in the teleportation protocol are mode-local as they are described in terms of operators relative to the fixed spatial locations *Y* and *L* on the one hand and *R* on the other. The main difference with respect to the metrological setting discussed in the previous section is that the analysis of the teleportation protocol needs the extension of the two-mode subalgebra, $A_{L}$, to a four-mode one, $A_{YL}={\left\{a_{Y,\sigma },a_{Y,\sigma }^{†},a_{L,\sigma },a_{L,\sigma }^{†}\right\}}_{\sigma =↑,↓}$.

The teleportation performances are measured by the fidelity given by the overlap between the input state |Φ〉 and the obtained state of the modes (X,↓) and (R,↑) (as if they were in the same Hilbert space) averaged over all Bell measurement outcomes and over all |Φ〉. For two-mode boson states, the teleportation fidelity turns out to be [74,75]:

(42) $$\begin{array}{ccc} & & f(|\Psi \rangle )=\frac{2}{M+2}\left(1+\sum_{k\neq j,\;k,j=0}^{N}\frac{\max\{0,M+1-|k-j|\}}{2M+2}\;\times \right.\\ & & \;\left.\times \;\frac{\langle \mathrm{vac}|a_{R,↓}^{N-k}a_{L,↑}^{k}\left|\Psi \rangle \langle \Psi \right|{\left(a_{L,↑}^{†}\right)}^{k}{\left(a_{R,↓}^{†}\right)}^{N-j}|\mathrm{vac}\rangle }{\sqrt{k!(N-k)!j!(N-j)!}}\right). \end{array}$$

If the resource state is $|\Psi_{\mathrm{unif}}\rangle$, the final state of modes (X,↑) and (R,↓) is the same as the state |Φ〉 in (25) for a fraction (N−M+1)/(N+1) of measurement outcomes, while other outcomes result in teleporting only some components of the same state. Thus, the teleportation fidelity approaches one for a large particle number in the resource state:

(43) $$f(|\Psi_{\mathrm{unif}}\rangle )=1-\frac{M}{3N+3}.$$

Teleportation is not exact for all Bell measurement outcomes because of the particle number conservation. When the resource state is $|\Psi_{\mathrm{coh}}\rangle$ with ξ=1/2, the teleportation fidelity also approaches one when N→∞:

(44) $$f\left(|\Psi_{\mathrm{coh}\rangle }\right)=1-O\left(\frac{M^{2}}{N}\right).$$

It is remarkable that coherent states $|\Psi_{\mathrm{coh}}\rangle$ provide very good teleportation performances with negligible distortions for large *N*, although they behave classically for the estimation of interferometric phases. Instead, the resource state $|\Psi_{\ell_{1},\ell_{2}}\rangle$ gives rise to poor teleportation performances, very close to those of mode-separable states $|\Psi_{\mathrm{Fock}}\rangle$ in (23), which yields:

(45) $$f\left(|\Psi_{\mathrm{Fock}\rangle }\right)=\frac{2}{M+2}.$$

This fidelity equals the maximum fidelity for teleporting (M+1)-level distinguishable particles; further, note that also the mode (Y,↓) to be teleported by means of the protocol discussed here has M+1 orthogonal states. The case of N=M=1 is particularly suited to enlighten how mode entanglement is able to capture the role of indistinguishability. Indeed, in such a case, the states |Ψ〉 and |Φ〉 are single-particle states, and the Bell-like measurement outcomes that provide perfect teleportation are associated with projections onto single-particle states. It is therefore clear that correlations with respect to the mode-algebraic bi-partitions, rather than among particles, is the relevant resource (see Appendix B).

The above protocol can also be applied to swap entanglement: the modes (X,↑) and (R,↓) in the initial state are not entangled, while they are after the protocol. If the aim is to distribute as much entanglement as possible, a measure of the entangled shared by the modes (X,↑) and (R,↓) is a better figure of merit than the teleportation fidelity as the latter carries information that is not relevant for the entanglement distribution [74,75].

## 4. Discussion

We showed that, in two specific quantum metrological and teleportation tasks based on systems of indistinguishable particles, mode entanglement is the quantum resource that allows for enhanced performances. Indeed, mode entanglement is the natural generalization to identical particles of the notion of entanglement of distinguishable particles; it provides a useful resource to beat the limitations due to local operations and classical communication. According to the discussion at the end of Section 2, mode-separable state vectors with respect to an algebraic bipartition remain separable under the action of operations that are mode-local with respect to the same bipartition, while classical communications can only generate statistical mixtures of states resulting from local operations [76].

The notion of locality that appears very naturally in the second quantization context cannot be consistently formulated in other approaches to indistinguishable particle entanglement [48,50]. It is therefore interesting to compare the performances of the above quantum information tasks within other entanglement theories. In particular, we considered two different approaches to identical particle entanglement that are based not on the notion of modes, rather than on that of particles.

### 4.1. No-Label Approach

The no-label approach characterizes the entanglement of *N* indistinguishable particles with at least two degrees of freedom within a formalism that avoids the use of unphysical particle labels [77,78]; as a consequence, state vectors of particles with only one degree of freedom turn out to be always unentangled. We adopted a reformulation in second quantization that is more convenient especially for many-particle states (see [48,78]). In this approach, the entanglement shared between *n* particles and the remaining N−n ones in an *N*-boson state vector |Ψ〉 is (i) defined with respect to a given choice of a finite-dimensional subspace K of the single-particle Hilbert space and (ii) evaluated by means of the von Neumann entropy of a matrix $\rho^{\left(n\right)}$ that is derived from the *N*-particle projector |Ψ〉〈Ψ| as follows. An orthonormal basis $\{|\psi_{j}\rangle {\}}_{j=1}^{p}$ is selected in the subspace K; then, a selective measurement is performed based on the set of operators $\prod_{j=1}^{p}a_{\psi_{j}}^{n_{j}}$ with all possible choices of occupation numbers of the corresponding modes such that $\sum_{j=1}^{p}\;n_{j}=n$, transforming |Ψ〉〈Ψ| into:

(46) $$\rho^{\left(n\right)}=\frac{\sum_{n_{1}+\cdots n_{p}=n}\left(\prod_{j=1}^{p}a_{\psi_{j}}^{n_{j}}\right)\left|\Psi \rangle \langle \Psi \right|\left(\prod_{k=1}^{p}{\left(a_{\psi_{k}}^{n_{k}}\right)}^{†}\right)}{\sum_{n_{1}+\cdot +n_{p}=n}\langle \Psi |\prod_{j=1}^{n}{\left(a_{\psi_{j}}^{n_{j}}\right)}^{†}a_{\psi_{j}}^{n_{j}}|\Psi \rangle }\;.$$

As regards $\rho^{\left(n\right)}$, two facts need be noticed:

1. while the von Neumann entropy of $\rho^{\left(n\right)}$ does depend on the chosen subspace K, it does not depend on the chosen orthonormal basis of K;
2. the operators $\prod_{j=1}^{p}a_{\psi_{j}}^{n_{j}}$ remove *n* particles from the *N*-particle state |Ψ〉. Therefore, $\rho^{\left(n\right)}$ is supported by the Fock sector with N−n particles; however, it is not an N−n-particle reduced density matrix as would result from a partial trace with respect to the discarded degrees of freedom: for instance, it does not reproduce the expectations of (N−n)-particle observables [49].

In order to make a comparison between the mode and the no-label approaches to identical particle entanglement in the case of the metrological task, let K be the two-dimensional subspace of $C^{4}$ spanned by the vectors |L,↓〉 and |L,↑〉. This latter is the usual choice in the applications of the no-label approach, which aims at addressing quantum correlations between localized particles. The matrix $\rho^{\left(n\right)}$ results then:

(47) $$\rho^{\left(n\right)}=\frac{\sum_{k=0}^{n}a_{L,↑}^{k}\;a_{L,↓}^{n-k}\;|\Psi \rangle \langle \Psi |\;{\left(a_{L,↓}^{n-k}\right)}^{†}\;{\left(a_{L,↑}^{k}\right)}^{†}}{\sum_{k=0}^{n}\langle \Psi |{\left(a_{L,↑}^{k}\right)}^{†}a_{L,↑}^{k}\;{\left(a_{L,↓}^{n-k}\right)}^{†}a_{L,↓}^{n-k}|\Psi \rangle }\;.$$

In the case of states |Ψ〉 of the form (25), one finds:

(48) $$a_{L,↑}^{k}\;a_{L,↓}^{n-k}\;|\Psi \rangle =\delta_{kn}\sum_{\ell =n}^{N}\alpha_{\ell }\;\frac{a_{L,↑}^{n}\;{\left(a_{L,↑}^{†}\right)}^{\ell }}{\sqrt{\ell !}}\frac{{\left(a_{R,↓}^{†}\right)}^{N-\ell }}{\sqrt{(N-\ell )!}}\;|\mathrm{vac}\rangle \;,$$

whence:

(49) $$\rho^{\left(n\right)}=|\Psi^{\left(n\right)}\rangle \langle \Psi^{\left(n\right)}\left|\;,\;\right|\Psi^{\left(n\right)}\rangle =\frac{1}{\sqrt{\sum_{\ell =n}^{N}{\left|\alpha_{\ell }\right|}^{2}}}\;\sum_{\ell =n}^{N}\alpha_{\ell }\;\frac{{\left(a_{L,↑}^{†}\right)}^{\ell -n}}{\sqrt{(\ell -n)!}}\;\frac{{\left(a_{R,↓}^{†}\right)}^{N-\ell }}{\sqrt{(N-\ell )!}}\;|\mathrm{vac}\rangle \;.$$

In this case, the matrix $\rho^{\left(n\right)}$ is thus a pure state, and therefore, its von Neumann entropy is zero for any *n*. As a consequence, according to the no-label approach, the states |Ψ〉 are not entangled with respect to any particle partitioning.

Within the no-label approach, as much as the notion of entanglement, also that of locality depends on the single-particle subspace K. In particular, single-particle operators whose measurement projects single-particle states onto K are deemed local [77,79]. Then, it follows that the unitary operator $U_{\theta }$ in (29) that implements the action of the interferometer discussed in the metrological context is local the product of the two spatially local phase-shifts in (31). The puzzle here is thus as follows: in the mode entanglement approach, the metrological quantum advantage is due to the entanglement of the state relative to the algebraic bipartition with respect to which the interferometer action is instead local. On the contrary, in the no-label approach, neither the state is entangled nor the interferometer is non-local, yet the quantum advantage is there as predicted by the behavior of the Fisher information, which is only due to the structure of the state |Ψ〉 and of the unitary operator $U_{\theta }$. Experimental evidence that the shot-noise limit is beaten would then force one to accept that, in the no-label approach, quantum metrological advantages might occur without either quantum entanglement or quantum non-locality during any step of the protocol.

Analogously, in the no-label approach, operators of the form:

(50) $$𝟙⊗O_{L}\;+\;O_{L}⊗𝟙\;\mathrm{with}\;O_{L}:=|L\rangle \langle L|\;O$$

and *O*, a single particle operator, in first quantization, or:

(51) $$\sum_{\sigma ,\sigma^{\prime }=↑,↓}\langle \sigma |O|\sigma^{\prime }\rangle \;a_{L,\sigma }^{†}\;a_{L,\sigma^{\prime }}\;,$$

in second quantization, are local. Such structures can be straightforwardly generalized so that *n*-particle local operators have the form:

(52) $$\sum_{\sigma_{j},\sigma_{j}^{\prime }=↑,↓}\langle \sigma_{1}|⊗\langle \sigma_{2}|\cdots ⊗\langle \sigma_{n}\left|O\right|\sigma_{1}^{\prime }\rangle ⊗|\sigma_{2}^{\prime }\rangle \cdots ⊗|\sigma_{n}^{\prime }\rangle \prod_{j=1}^{n}a_{L,\sigma_{j}}^{†}a_{L,\sigma_{j}^{\prime }}.$$

Therefore, the measurements in the teleportation protocol involve local observables in the *L* and *Y* positions, and thus act locally on the states |Ψ〉. Furthermore, also the last step of the teleportation protocol is local as it acts unitarily with respect to the mode (R,↓) and possibly on nearby auxiliary modes. Like in the case of phase estimation with local interferometers, the teleportation protocol interpreted within the no-label approach is apparently implementable without either entanglement or quantum non-locality, quite a paradoxical conclusion.

### 4.2. Particle Entanglement: Another Approach

Another particle-based approach to entanglement in systems of indistinguishable particles, sometimes called particle entanglement, defines as separable states those where all particles are in the same single-particle state |ψ〉, namely ${|\psi \rangle }^{⊗N}$ in first quantization, or $\frac{{\left(a^{†}\right)}^{N}|\mathrm{vac}\rangle }{\sqrt{N!}}$ for any mode-creation operator $a^{†}$ in second quantization [80,81,82,83].

This approach is a straight extension of the standard theory of entanglement to identical particles. Indeed, unlike in Section 2, symmetrization is taken as a legitimate source of entanglement. For instance, in this way, all Fermionic state vectors turn out to be entangled. Within this approach, we show that quantum advantages as teleportation emerges without particle entanglement, for they are obtainable from particle-separable states. It then follows that this particular type of particle entanglement is not consistent with the operational point of view that asks for entanglement in order to achieve quantum advantages.

As a first example, let us consider the teleportation protocol discussed in Section 3.2, and set N=M=1. Then, the resource state (25) reduces to $|\Psi \rangle =\left(a_{L,↑}^{†}+a_{R,↓}^{†}\right)|\mathrm{vac}\rangle$, and thus to the states $|\Psi_{\ell_{1}=0,\ell_{2}=1}\rangle$, $|\Psi_{\mathrm{unif}}\rangle$ and $|\Psi_{\mathrm{coh}}\rangle$ in (26)–(28) with N=1. The state |Ψ〉 is mode-entangled, but as a single-particle state, it is trivially not particle-entangled. On the other hand, the protocol uses projectors onto single-particle states that provide probabilistically perfect teleportation, but, as such, cannot generate particle entanglement. Therefore, the particle entanglement so far considered is not necessary for teleportation.

As a second instance where paradoxical results emerge adopting the above definition of particle entanglement, let us consider a slightly different teleportation protocol that aims at distributing entanglement at distant locations *X* and *R*, as done in entanglement swapping. The protocol hinges upon an input state of coherent form,

(53) $$|\Phi_{\mathrm{coh}}\rangle =\frac{1}{\sqrt{M}}{\left(\sqrt{\zeta }\;a_{X,↑}^{†}+e^{i\vartheta }\sqrt{1-\zeta }\;a_{Y,↓}^{†}\right)}^{M}|\mathrm{vac}\rangle ,$$

and upon the resource state $|\Psi_{\mathrm{coh}}\rangle$ as in (28). Both of these states are particle-separable according to the above definition of particle entanglement. Let us take as the protocol global initial state the combination $|\Phi_{\mathrm{coh}}\rangle |\Psi_{\mathrm{coh}}\rangle$; notice that this state is not particle-separable since it is not the product of the same single-particle states, despite the fact that $|\Phi_{\mathrm{coh}}\rangle$ and $|\Psi_{\mathrm{coh}}\rangle$ can be prepared independently at distant locations without any interactions between the corresponding particles. In addition, let us replace the Bell-like measurement with the following generalized measurement:

(54) $$\rho ⟶\frac{E_{i}\rho E_{i}^{†}}{\mathrm{Tr}(\rho E_{i}^{†}E_{i})}\;,$$

with outcomes i∈{0,1} and:

(55) $$\begin{array}{cc} E_{0} & =|\eta ,\omega \rangle \langle \eta ,\omega |\;,\;|\eta ,\omega \rangle =\frac{1}{\sqrt{n}}{\left(\sqrt{\eta }\;a_{Y,↓}^{†}+e^{i\omega }\sqrt{1-\eta }\;a_{L,↑}^{†}\right)}^{n}|\mathrm{vac}\rangle \;, \end{array}$$

(56) $$\begin{array}{cc} E_{1} & =𝟙-E_{0}\;, \end{array}$$

where 0≤η≤1 and ω an arbitrary phase. Note that only $E_{1}$ can generate entanglement as $E_{0}$ projects onto a particle-separable state. Nevertheless, by discarding the “1” outcome and concentrating on the “0” one, as the outcome of the protocol, one ends up with the state:

(57) $$\frac{E_{0}|\Phi_{\mathrm{coh}}\rangle |\Psi_{\mathrm{coh}}\rangle }{\sqrt{\langle \Phi_{\mathrm{coh}}|\langle \Psi_{\mathrm{coh}}|E_{0}^{†}E_{0}|\Phi_{\mathrm{coh}}\rangle |\Psi_{\mathrm{coh}}\rangle }}\;,$$

with probability:

(58) $$\langle \Phi_{\mathrm{coh}}|\langle \Psi_{\mathrm{coh}}|E_{0}^{†}E_{0}|\Phi_{\mathrm{coh}}\rangle |\Psi_{\mathrm{coh}}\rangle \;.$$

For N=M=n, one explicitly finds:

(59) E0|Φcoh〉|Ψcoh〉==|η,ω〉1N!∑k=0NNk2η(1−ξ)(1−ζ)ei(ϑ+φ)N−k2

(60) $$\begin{array}{cc} \; & \;\times {\left(\xi \zeta \left(1-\eta \right)e^{-i\omega }\right)}^{\frac{k}{2}}{\left(a_{X,↑}^{†}\right)}^{k}{\left(a_{R,↓}^{†}\right)}^{N-k}|\mathrm{vac}\rangle . \end{array}$$

This final state turns out to be in general entangled with respect to the untouched modes (X,↑) and (R,↓). Therefore, the above swapping protocol probabilistically generates entanglement starting from a state, $|\Phi_{\mathrm{coh}}\rangle |\Psi_{\mathrm{coh}}\rangle$, which, as already stressed, can be prepared using only local operations, quite a striking paradox.

Such an inconsistency originates from the impossibility of reconciling the above definition of particle entanglement with any underlying notion of identical particle locality (see Remarks 1 and 3).

A resource theory associated with the notion of particle entanglement here discussed has been recently proposed [83]. In general, resource theories describe quantum resources beyond entanglement [10]; they are based on defining resourceless states, called free states, and resourceless operations, called free operators, as a subset (sometimes a proper subset as in the case of resource theories of entanglement [76,84] and quantum coherence [85]) of operators that cannot transform free states into resourceful ones. It turns out that the resource theory proposed in [83] identifies states of the form $|\Phi_{\mathrm{coh}}\rangle |\Psi_{\mathrm{coh}}\rangle$ as resourceful, but without specifying any notion of particle-local operations. Instead, in discussing entanglement theory, the notion of locality is crucial in the definition of free operations, which typically consist of local operations and classical communication [76].

## 5. Conclusions

We discussed a fully consistent generalization of entanglement theory to systems of indistinguishable particles and some of its applications to quantum information processing. Such a general theory, namely mode entanglement, is formulated in second quantization and accounts for quantum correlations between modes in the Fock space. Indeed, identical particles are not individually addressable, unless they are effectively distinguished by unambiguous properties, i.e., orthogonal states of certain degrees of freedom. On the other hand, modes are experimentally addressable [37,38,39], and mode addressability retrieves particle addressability when identical particles become effectively distinguishable [48]. In general, however, full physical consistency requires that the relevant degrees of freedom in many-body quantum information processing be modes rather than particles.

We showed that mode entanglement is the relevant quantum resource for interferometric phase estimation and teleportation, two benchmark protocols that are central in quantum technologies. Indeed, indistinguishability improves the capability of these protocols with respect to those making use of distinguishable particles. First of all, identical particles can be distinguished at the cost of sacrificing certain degrees of freedom that can only be used as particle labels and not as an active part of information processing. Secondly, indistinguishable particles lift off the load to generate entanglement at the input of an interferometer in order to estimate the phase with quantum-enhanced accuracy, the reason being that indistinguishability allows the interferometer itself to generate mode entanglement. Finally, mode entanglement in systems of indistinguishable particles enables the implementation of mode teleportation that thus generalizes standard teleportation.

We also compared the above protocols with alternative approaches to indistinguishable particle entanglement within the context of resource analysis, pointing out logical contradictions that arise in adopting those definitions. In particular, the no-label approach that introduces an ad hoc formalism in order to avoid unphysical particle labels attributes no role to entanglement in the realization of the protocols described above. Instead, the other discussed approach to particle entanglement attributes entangling power to the symmetrization of identical particle pure states. As such, it entails that teleportation can be used to generate entanglement between distant particles using only non-entangling operations.

These results clearly pinpoint internal inconsistencies in those definitions of “particle entanglement”, making them unreliable in their physical predictions. Instead, in all so-far tested physical applications, no inconsistencies emerge using second-quantized mode entanglement in describing quantum correlations in system of identical particles: indeed, this approach is able to correctly identify entanglement as a crucial ingredient in the realization of quantum protocols.

We hope that these findings will stimulate further research on the use of many-body systems consisting of identical constituents in quantum information processing, and in particular encourage the development of quantum technological advances able to experimentally confirm the above discussed results.

## Data Availability

Not applicable.

## Appendix A. Bell-Like Measurement

The Bell-like measurement in the teleportation protocol is a generalization of that used in the teleportation of qudits [70]. It consists of projectors onto the following states:

(A1) $$|B_{\ell ,\lambda }\rangle_{YL}=\sum_{k=0}^{M}\frac{e^{2\pi i\frac{\lambda k}{M+1}}}{\sqrt{M+1}}\;\frac{{\left(a_{Y,↓}^{†}\right)}^{M-k}}{\sqrt{(M-k)!}}\cdot \frac{{\left(a_{L,↑}^{†}\right)}^{k+\ell }}{\sqrt{(k+\ell )!}}\;|\mathrm{vac}\rangle \;,$$

where ℓ∈{0,1,⋯,N−M} and λ∈{0,1,⋯,M}.

The formal difference with respect to the Bell measurement for qudit teleportation can be appreciated by re-writing:

$${\left(a_{Y,↓}^{†}\right)}^{M-k}{\left(a_{L,↑}^{†}\right)}^{k+l}|\mathrm{vac}\rangle =\sqrt{(M-k)!(k+l)!}\;{|M-k,k+l\rangle }_{YL}\;.$$

Indeed, the sum k+ℓ is taken modulo *N* for qudits, and the dimensions of each qudit Hilbert space are the same, while, in the Fock space, the qudit measurement violates the conservation of the particle number that is imposed here. In order to overcome this difficulty, we consider the standard sum k+ℓ and allow for larger occupation numbers of the mode (L,↑). Despite this generalization, the states (A1) do not span the state space available for the modes (Y,↓) and (L,↑), and the corresponding measurement is not complete. Nevertheless, the ratio between the space spanned by (A1) and the required space of the modes (Y,↓) and (L,↑) is $\frac{N-M+1}{N+1}$ approaching one for N≫M. Furthermore, measurements with outcomes (ℓ,λ) projecting onto states (A1) provide perfect teleportation when using the resource state $|\Psi_{\mathrm{unif}}\rangle$.

A complete measurement including projectors onto (A1) is a projective measurements onto the following states:

(A2) $$\begin{array}{cc} \; & |B_{\ell ,\lambda }\rangle_{YL}=\sum_{k=\max\{0,-\ell \}}^{\min\{M,N-\ell \}}\frac{e^{2\pi i\frac{\lambda k}{C_{\ell }}}}{\sqrt{C_{\ell }}}\;\frac{{\left(a_{Y,↓}^{†}\right)}^{M-k}}{\sqrt{(M-k)!}}\cdot \frac{{\left(a_{L,↑}^{†}\right)}^{k+\ell }}{\sqrt{(k+\ell )!}}\;|\mathrm{vac}\rangle ,\\ \; & \\ \; & C_{\ell }=\left\{\begin{array}{cc} M+\ell +1 & \mathrm{if}-M⩽\ell <0\\ M+1 & \mathrm{if}0⩽l⩽N-M\\ N-\ell +1 & \mathrm{if}N-M<\ell ⩽N \end{array}\right.,\\ \; & \\ \; & \ell \in \left\{-M,-M+1,\cdots ,N\right\},\;\lambda \in \left\{0,1,\cdots ,C_{\ell }-1\right\}. \end{array}$$

Projectors onto states (A2) not included in (A1) result in the teleportation only of some components of input states |Ψ〉 and slightly increase the teleportation fidelity.

## Appendix B. Teleportation for Distinguishable Particle

For a better comparison with the case of a distinguishable particle, it is instructive to set N=M=1 and rephrase the teleportation protocol presented in the previous Appendix within the first quantization approach. The input and the resource states are, respectively,

(A3) $$|\Phi \rangle =c_{0}|X,↑\rangle +c_{1}|Y,↓\rangle \in H_{\mathrm{input}},\;|\Psi \rangle =\alpha_{0}|R,↓\rangle +\alpha_{1}|L,↑\rangle \in H_{\mathrm{resource}},$$

and the Bell-like measurement outcomes that provide perfect teleportation correspond to projections onto the states:

(A4) $$|B_{\pm }\rangle =\frac{|Y,↓\rangle \pm |L,↑\rangle }{\sqrt{2}}.$$

Whether these projectors act either on the input state or on the resource state, it is not possible to reproduce the desired output state, namely:

(A5) $$c_{0}|X,↑\rangle +c_{1}|R,↓\rangle .$$

Indeed, standard teleportation protocols for distinguishable particle states requires two- (or many-) particle resource states and Bell measurements. Teleportation in the framework of mode entanglement recovers the standard protocol for a fixed particle number in locations *Y*, *L*, and *R* [48,74,75]. An example is provided by the following input state and resource state:

(A6) $$\begin{array}{ccc} |\Phi \rangle & = & c_{0}|Y,↑\rangle +c_{1}|Y,↓\rangle \in H_{\mathrm{input}}\;, \end{array}$$

(A7) $$\begin{array}{ccc} |\Psi \rangle & = & \frac{|L,↓\rangle ⊗|R,↓\rangle +|L,↑\rangle ⊗|R,↑\rangle }{\sqrt{2}}\in H_{\mathrm{resource}}\;, \end{array}$$

and by projectors onto the following states:

(A8) $$\begin{array}{ccc} |B_{\pm }\rangle & = & \frac{|Y,↓\rangle ⊗|L,↓\rangle \pm |Y,↑\rangle ⊗|L,↑\rangle }{\sqrt{2}}\;, \end{array}$$

(A9) $$\begin{array}{ccc} |B_{\pm }^{\prime }\rangle & = & \frac{|Y,↓\rangle ⊗|L,↑\rangle \pm |Y,↓\rangle ⊗|L,↑\rangle }{\sqrt{2}}. \end{array}$$
